# 1,2,4-Triazine-based Materials: Spectroscopic Investigation, DFT, NBO, and TD-DFT Calculations as Well As Dye-sensitized Solar Cells Applications

**DOI:** 10.1007/s10895-022-03005-1

**Published:** 2022-07-21

**Authors:** Mahmoud A. S. Sakr, Maram T. H. Abou Kana

**Affiliations:** 1 Chemistry Department, Center of Basic Science, Misr University for Science and Technology (MUST), 6Th of October City, Egypt; 2 Laser Sciences and Interactions Department, National Institute of Laser-Enhanced Sciences (NILES), Cairo University, Giza, Egypt

**Keywords:** Solar cell, Dye-sensitized solar cells (DSSCs), Bis (5,6-diphenyl-1,2,4-triazines), Optical properties, Density functional theory (DFT), Time dependent density functional theory (TD-DFT)

## Abstract

In this manuscript, we report four series for 1,2,4-triazine derivatives as dye-sensitized solar cells (DSSCs). Density functional theory (DFT) methods via utilizing Becke's three-parameter functional and LeeeYangeParr functional (B3LYP) level with 6-31G (d, p) basis set to investigate their modeling molecular structures. Optimized molecular structures for studied molecular structures are obtained using the DFT/B3LYP/6-31G (d, p) method. In addition, the time-dependant density functional theory (TD-DFT) is used to study the optoelectronic properties and absorption spectra using DFT/CAM-B3LYP/ 6-31G +  + (d, p) level in the Gaussian 09 program. The highest occupied molecular orbital (HOMO), lowest unoccupied molecular orbital (LUMO), energy gap (E_g_), light harvest efficiency (LHE), and open-circuit voltage (Voc) of the studied molecular structures are calculated and illustrated. These properties indicate that these molecular modeling structures as good candidates for utilization in organic DSSCs.

## Introduction

Because of the great challenge used in new research on renewable energy sources, solar cells are considered one of the most important renewable energies recently [1–4]. Photovoltaic technologies have become one of the most important topics in solar cells to convert the sun into electrical energy [5, 6]. In addition, the most important challenges are represented in capturing solar energy and converting it into electrical energy at a low cost [7]. It was taken out of the photovoltaic devices that are based on inorganic materials in their manufacture, such as crystalline and amorphous silicon, cadmium telluride (CdTe), gallium arsenide (GaAs), with the knowledge that they give their efficiency from 10 to 32% [8]. However, since these are expensive materials and scarce, in addition to their toxicity, many researchers have resorted to searching for other new, cheap, and more efficient materials. According to what will be said, solar cells based on organic compounds are considered an attractive and appropriate choice due to their flexibility and ease of processing in addition to their low cost, but their efficiency at present is considered less than those that depend on inorganic materials [9]. The efficiency obtained from these types of cells is still not marketable, as the most efficient devices are 4 to 5% [10].

Recently, new organic compounds used in solar cells have been studied and developed. Among these materials are the dyes for sensitive solar cells (DSSCs), as they receive great interest among researchers due to their low cost and high efficiency in converting solar energy into electricity [11]. Moreover, the manufacture of their devices is easy. Also, photovoltaic cells based on the DSSCs have many advantages, including their compatibility with many supporting materials and production under moderate conditions that make them less expensive compared to other DSSCs [12, 13]. The first DSSCs were based on titanium dioxide, which was discovered in 1991, and their efficiency was from 7 to 8 percent. [13].

In this manuscript, the results of quantum calculations for four organic compounds based on 1,2,4-triazine derivatives as dye-sensitized solar cells (DSSCs) are presented. In these structures, we used sulfur atom as electron donor unit (D) and the phenyl and triazine groups were used as electron acceptor groups (A) for all compounds.

Time-Dependent DFT (TD-DFT) has been widely used to discuss the properties of organic compounds in their excited state because its high accuracy is reasonable with the ab-initio method and less computational time cost. In this paper, the obtained calculated results via utilizing TD-DFT/ CAM-B3LYP/ 6–31 +  + G (d, p) like the optoelectronic properties and photovoltaic properties (open-circuit voltage (Voc), oxidation potential energy, and electron injection force) of all molecular structures were investigated and presented.

## Computational Investigation

The molecular modeling and photoelectronic properties of the studied compounds (DTT, BDTTB, BDTTMB, and BDTTMP) based on 1,2,4-triazine were obtained and investigated using Density functional theory (DFT) methods via utilizing Becke's three-parameter functional and LeeeYangeParr functional (B3LYP) [14] level with 6-31G(d, p) [15] basis set. All computational calculations were presented by utilizing the Gaussian 09 program [15]. Upon utilizing the DFT/B3LYP/6-31G (d, p) level, the molecular structures of neutral molecules are optimized, and their molecular electronic properties as HOMO, LUMO levels, and the energy gap (E_g_) are obtained. The oscillator strengths (f) and the optical transitions have been illustrated using TD-DFT combined with CAM-B3LYP functional and 6- 31G +  + (d, p) basis set. Finally, the ultraviolet–visible (UV–Vis.) absorption spectra of the studied molecules were presented utilizing Gauss View software [16].

## Result and Discussions

### Studied Molecular Structures

**Fig. 1:**
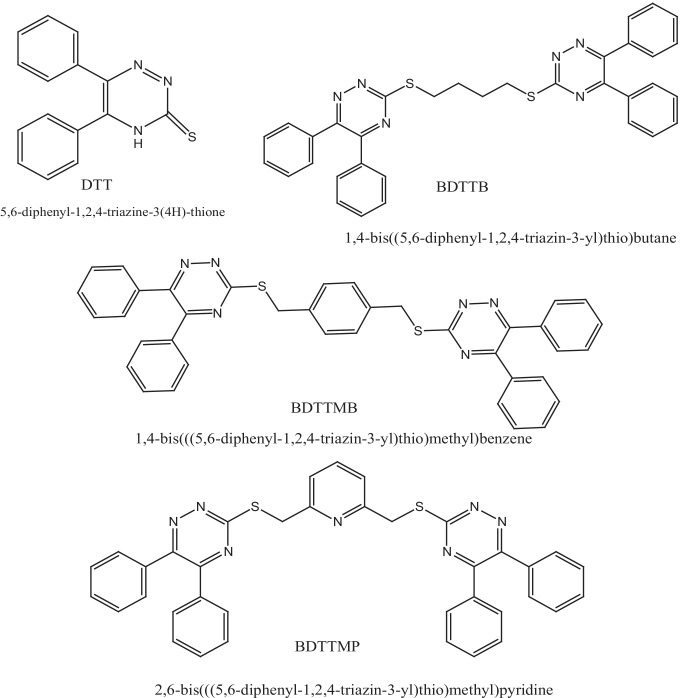
Studied molecular structures DTT, BDTTB, BDTTMB and BDTTMP

The studied molecular structures as shown in Fig. 1 are 5, 6-diphenyl-1,2,4-triazine-3(4H)-thione (DTT), 1,4-Bis((5,6-diphenyl-1,2,4-triazin-3-yl)thio)butane (BDTTB), 1,4-Bis(((5,6-diphenyl-1,2,4-triazin-3-yl)thio)methyl)benzene (BDTTMB) and 2,6-Bis(((5,6-diphenyl-1,2,4-triazin-3-yl)thio)methyl)pyridine (DTTMP). As shown in Fig. 1, the organic chemical compounds BDTTB, BDTMP and BDTMP consist of two main DTT groups, while the intermediate group differs from one compound to the other. Since in BDTTB the middle group is a butyl group, and in the BDTTMB compound, the butyl group is replaced by a p-xylenyl group, and finally in the BDTTMP compound, the middle group is 2,6-butidienyl. The four studied molecular structures under study were prepared, characterized and published in 2121 by Sakr et. al. [17].

### Molecular Structure Properties

**Fig. 2:**
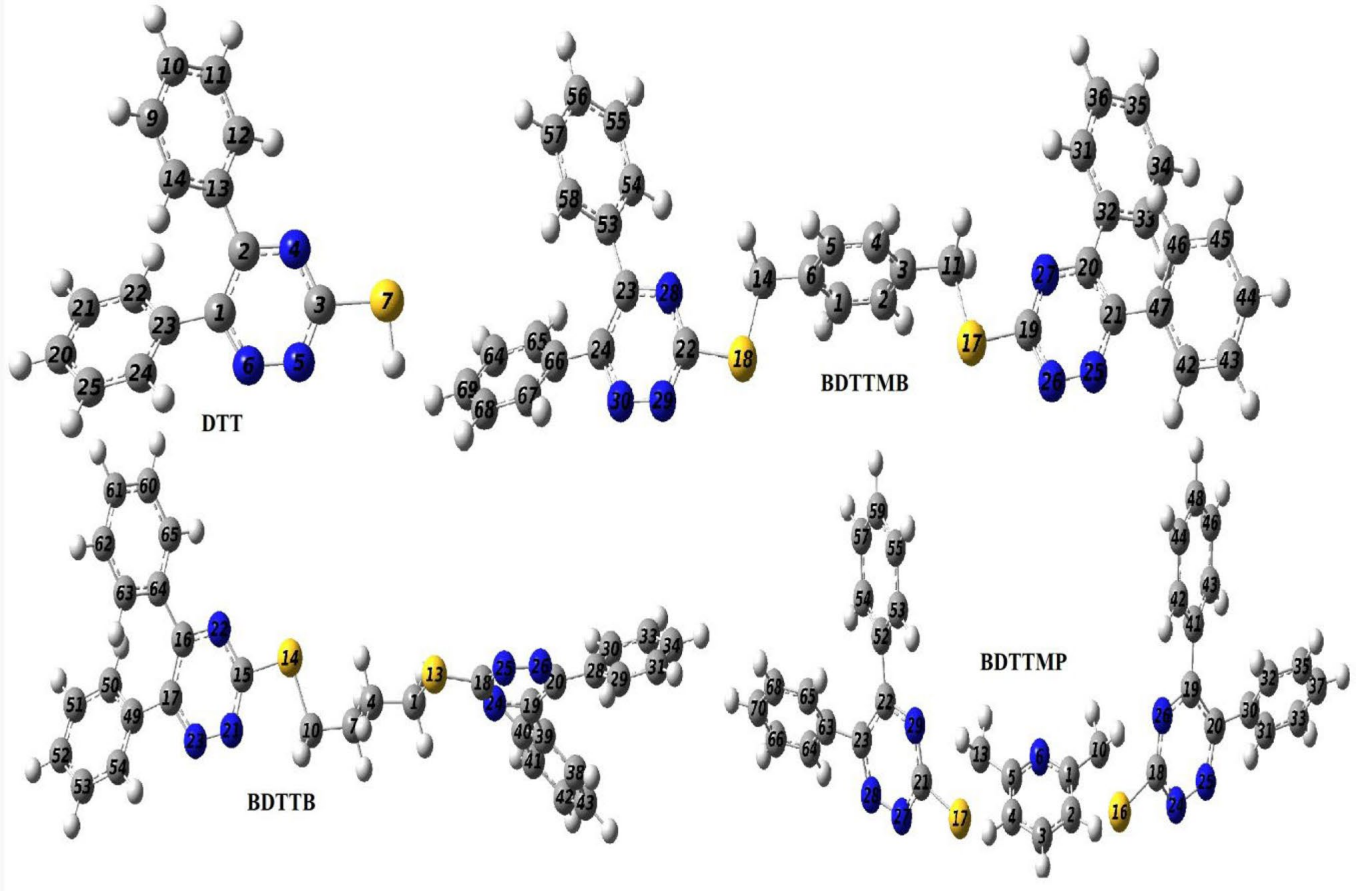
Optimized molecular structures for studied molecular structures using the DFT/B3LYP/6-31G (d, p) method

The optimization of the molecular structures under study has been obtained via using the DFT/B3LYP/6-31G (d, p) method in the gas state; the result of optimized molecular structures are presented in Fig. 2. The effect of substituents such as butyl in BDTTB, p-xylenyl in BDTTMB, and 2,6-butidienyl in BDTTMP has been studied on some important selected optimized molecular structure parameters such as (bond length in Å and dihedral angle in ^o^) for DTT optimized molecular structure; the results are collected in Table 1. Some important comments that can be deduced are as follows; (1) The DDT molecular structure is not planar as one of the two phenyl groups rotate out of the triazine by angle 33.44° to prevent the steric hindrance. (2) all molecular structures (BDTTB, BDTTMB, and BDTTMP) are not planar as one of the phenyl groups rotates by 34.04, 15.13, and 35.49° for BDTTB, BDTTMB, and BDTTMP respectively out of the triazine moiety to decrease steric hindrance. Hence, the π-interactions among sub-systems in each molecule are small. (3) Generally, the stability of molecular structure can be judged via the length of the bond [18], where the more stable molecular structure is combined with the shorter the bond length. As shown in Table 1, the bond lengths of DDT and BDTTMB are less than that of BDTTB and BDTTMP hence, the DDT and BDTTMB are more stable molecular structures compared to BDTTB and BDTTMP. (4) The selected bond angles for all studied molecular structures refer to SP^2^ hybridization.

**Table 1:** Selected optimized molecular structure parameters (bond length in Å and dihedral angle in ^o^) computed for DDT, BDTTB, BDTTMB, and BDTTMP in the gas phase using B3LYP/6-31G (d, p). For labeling, go to Fig. 2

DDT	Designation	BDTTB	Designation	BDTTMB	Designation	BDTTMP	Designation
C_2_-N_4_	1.336	C_16_-N_22_	1.342	C_23_-N_28_	1.339	C_22_-N_29_	1.356
N_4_-C_3_	1.332	N_22_-C_15_	1.337	N_28_-C_22_	1.332	N_29_-C_21_	1.339
C_3_-N_5_	1.342	C_15_-N_21_	1.348	C_22_-N_29_	1.347	C_21_-N_27_	1.358
N_5_-N_6_	1.325	N_21_-N_23_	1.328	N_29_-N_30_	1.318	N_27_-N_28_	1.344
C_1_-C_2_	1.426	C_17_-C_16_	1.429	C_24_-C_23_	1.421	C_23_-C_22_	1.424
C_2_-C_13_	1.483	C_16_-C_64_	1.484	C_23_-C_53_	1.484	C_22_-C_52_	1.480
C_1_-C_23_	1.484	C_17_-C_49_	1.485	C_24_-C_66_	1.484	C_23_-C_63_	1.480
C_3_-S_7_	1.772	C_15_-S_14_	1.767	C_22_-S_18_	1.770	C_21_-S_17_	1.816
C_12_-C_13_-C_2_-N_4_	33.44	C_65_-C_64_-C_16_-N_22_	34.04	C_54_-C_53_-C_23_-N_28_	148.55	C_53_-C_52_-N_22_-N_29_	35.49
C_13_-C_2_-C_1_-C_23_	15.04	C_64_-C_16_-C_17_-C_49_	14.36	C_53_-C_23_-C_24_-C_66_-	15.13	C_52_-C_22_-C_23_-C_63_	14.31
C_12_-C_13_-C_2_	119.00	C_15_-S_14_-C_12_-C_7_	177.56	C_22_-S_18_-C_14_-C_6_	176.67	C_21_-S_17_-C_13_-C_5_	179.48
C_13_-C_2_-N_4_	116.00	C_18_-S_13_-C_1_-C_5_	176.14	C_19_-S_17_-C_11_-C_3_	176.68	C_18_-S_16_-C_10_-C_1_	179.49
C_2_-N_4_-C_3_	117 0.00	C_16_-N_22_-C_5_	116.00	C_23_-N_28_-C_22_	117.00	C_22_-N_29_-C_21_	116.00
C_1_-N_6_-N_5_	121.00	C_17_-N_23_-N_21_	121.00	C_17_-N_23_-N_21_	121.00	C_23_-N_28_-N_27_	121.00
		C_65_-C_62_-C_16_	119.00	C_65_-C_62_-C_16_	121.00	C_53_-C_52_-C_22_	116.00

### Energy levels of the studied molecular structures

**Fig. 3:**
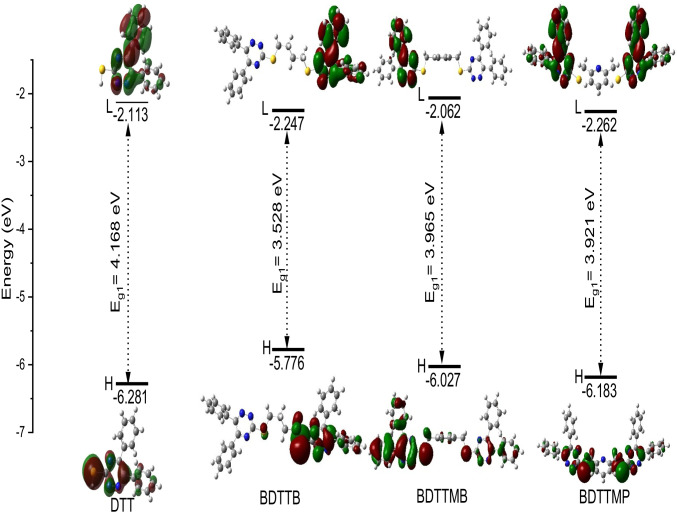
3 Graphical presentation of the highest occupied (HOMO), lowest unoccupied molecular (LUMO) orbitals, the energy of HOMO and LUMO, and energy gaps (E_g1_) for DTT, BDTTB, BDTTMB, and BDTTMP in gas at B3LYB/6-31G(d, p) level of theory

The graphical presentation of the HOMO and LUMO MOs, for DDT, BDTTB, BDTTMB, and BDTTMP compounds in gas at the B3LYB/6-31G (d, p) level of theory is shown in Fig. 3. It is noticeable from Fig. 3 that the HOMO MOs of the compounds under study are localized on certain parts of these compounds (sulfur atom, triazine, and phenyl groups), but not all of them. On the other side, the LUMO MOs are localized on (triazine and phenyl groups). This indicates that the sulfur atom, triazine, and phenyl groups act as a donor, but on the other hand, triazine and phenyl groups act as an acceptor. Furthermore, the calculated energy values of HOMO, HOMO-1, HOMO-2, LUMO, LUMO + 1, and LUMO + 2 and the energy gap between the following; (HOMO and LUMO (E_g1_), HOMO-1 and LUMO + 1 (E_g2_) and HOMO-2 and LUMO + 2(E_g3_)) of the compounds under study are written in Tables 2 and 3 The HOMO of the studied molecular structures are ordered as follows; DDT < BDTTMP < BDTTMB < BDTTB. Also, the LUMO energy MOs are arranged in the following order; BDTTMP < BDTTB < DTT < BDTTMB. The calculated E_g1_ of the studied molecular structures are arranged in the following order BDTTB < BDTTMP < BDTTMB < DTT. This indicates that compound BDTTB has the highest reactivity and compound DTT has the lowest reactivity. Also, the reduction in the E_g1_ value favors the red-shifted of the electronic absorption maximum peak in the UV–Vis absorption spectrum. Therefore, the electronic UV–Vis absorption spectra for BDTTB, BDTTMP, and BDTTMB are red shifted, in contrast to, the UV–Vis absorption spectrum of DTT. As a whole, the electron density of HOMO MOs is mainly located near the donor and the electron density of LUMO is mainly located near the acceptor. Consequently, the sulfur atom acts as an electron donor unit (D), and both the phenyl and triazine groups were used as electron acceptor units (A).

**Table 2:** Energy values of HOMO, HOMO-1, HOMO-2, LUMO, LUMO + 1, and LUMO + 2 and the energy gap between the following; (HOMO and LUMO (E_g1_), HOMO-1 and LUMO + 1 (E_g2_), HOMO-2 and LUMO + 2(E_g3_) for DTT, BDTTB, BDTTMB and BDTTMP compounds in gas at B3LYB/6-31G(d, p) level of theory

Compounds	E_HOMO_ (eV)	E_LUMO_ (eV)	E_g1_ (eV)	E_HOMO-1_ (eV)	E_LUMO+1_ (eV)	E_g2_ (eV)	E_HOMO-2_ (eV)	E_LUMO+2_ (eV)	E_g3_ (eV)
DTT	-6.281	-2.113	4.168	-6.469	-1.564	4.905	-6.920	-0.430	6.490
BDTTB	-5.776	-2.247	3.528	-6.106	-1.992	4.114	-6.286	-1.478	4.808
BDTTMB	-6.027	-2.062	3.965	-6.186	-2.160	4.026	-6.249	-1.626	4.623
BDTTMP	-6.183	-2.262	3.921	-6.045	-2.012	4.033	-6.300	-1.467	4.833

**Table 3:** Energy values of E_HOMO_, E_LUMO_, open-circuit voltage (Voc), and light-harvesting efficiency (LHE) by eV

Compounds	E_HOMO_ (eV)	E_LUMO_ (eV)	Voc (eV)	α (eV)	LHE(ev)
DTT	-6.281	-2.113	1.887	1.09	0.241
BDTTB	-5.776	-2.247	1.753	0.953	0.103
BDTTMB	-6.027	-2.062	1.938	1.138	0.042
BDTTMP	-6.183	-2.262	1.738	0.938	0.005

**Fig. 4:**
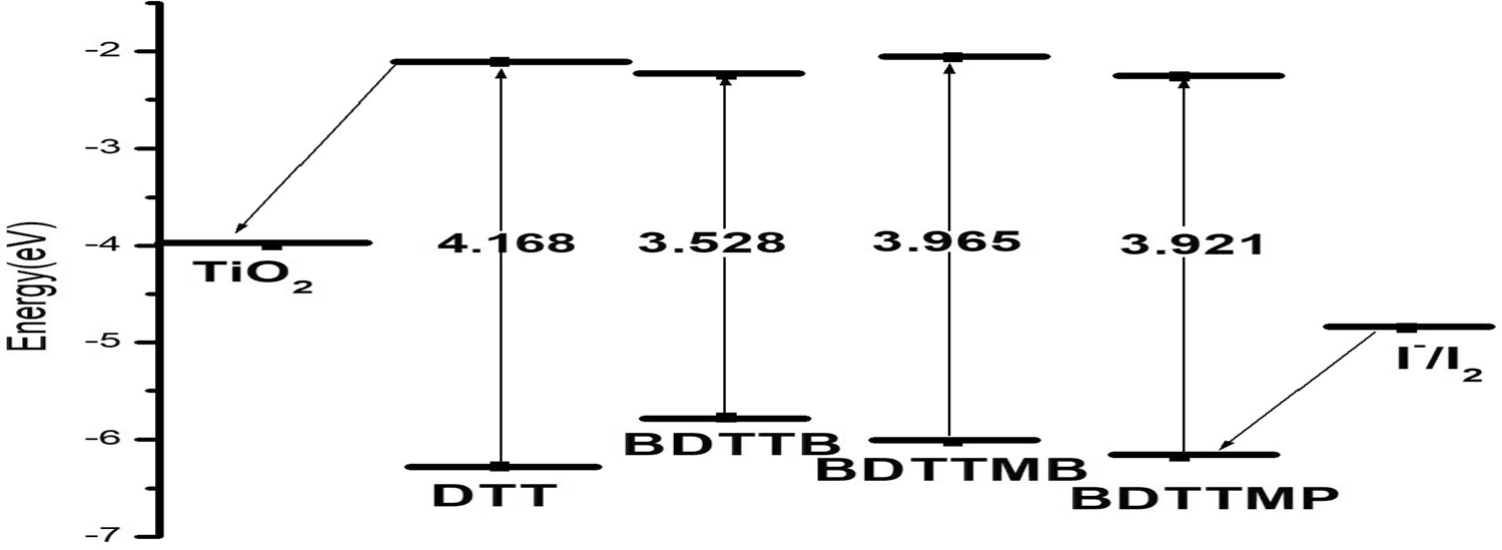
Frontier molecular orbital energies and energy gaps of TiO_2_, DTT, BDTTB, BDTTMB, BDTTMP, and I.^−^/I_2_

Figure 4 exhibits the molecular orbital energy levels of the four molecular structures DTT, BDTTB, BDTTMB, and BDTTMP in gas at the DFT/B3LYB/6-31G (d, p) level of theory. For the all-molecular structures under study, the HOMO energy is lower than the energy of I^−^/I_2_ (− 4.85 eV). This indicates that all studied compounds (DTT, BDTTB, BDTTMB, and BDTTMP) can more easily recover electrons from electrolytes (I_3_^−^). In addition to, the LUMO energy of the four studied molecules (DTT, BDTTB, BDTTMB, and BDTTMP) is higher than the conduction band energy of semiconductor TiO_2_ (− 4.00 eV) as shown in Fig. 4. Indicating, that the electrons can be successfully transferred into TiO_2_ from the excited state of all studied dyes. Consequently**,** all studied molecular structure dyes may be good candidates for application in photovoltaic devices.

The conversion efficiency (Ƞ) of sunlight to electrical energy in solar cell devices is determined by the short-circuit current density (Jsc), the open-circuit photovoltage (Voc), and the fill factor (FF) and incident solar power (Pinc). The Ƞ can be calculated by utilizing the following Eq. (1) [19]:

1 $$\eta =\frac{{\mathrm{J}}_{\mathrm{sc}}{\mathrm{V}}_{\mathrm{oc}}\mathrm{FF}}{{\mathrm{P}}_{\mathrm{inc}}}$$

From the formula, it can be seen that high V_OC_ and J_SC_ are the basis for producing photoelectric conversion efficiency. The maximum value for Voc is a significant photovoltaic parameter that can be obtained computationally via utilizing the difference between the HOMO of dye and the LUMO of the electron acceptor (conduction band of TiO_2_). The computational value of Voc has been calculated by utilizing the following (2) [20, 21]:

2 $${\mathrm{V}}_{\mathrm{oc}}=\left|{\mathrm{E}}_{\mathrm{HOMO}}^{\mathrm{donor}}\right|-\left|{\mathrm{E}}_{\mathrm{LUMO}}^{\mathrm{acceptor}}\right|-0.3$$

Whilst, in DSSCs, Voc can be approximately obtained as the different energy among LUMO of the dye and conduction band (CB) of the semiconductor

3 $$\mathrm{TiO}2\;({\mathrm E}_{\mathrm{CB}}=-4.0\mathrm{eV}):\;\;\;{\mathrm V}_{\mathrm{oc}}=\left|\mathrm E_{\mathrm{LUMO}}^{\mathrm{dye}}\right|-{\mathrm E}_{\mathrm{CB}}$$

The theoretically calculated value of Voc for all studied molecular structures is presented in Table 2. The range value of Voc is 1.738 to 1.938 eV for semiconductors (TiO_2_). These values are positive; thus indicating that the electron transfer will be easy from all studied molecular structures (DTT, BDTTB, BDTTMB, and BDTTMP) to TiO_2_. Furthermore, these values are sufficient to give the best efficient electron injection. Moreover, these triazine derivatives compounds can be utilized as sensitizers of the electron injection process from the excited dye to the conduction band of TiO_2_.

Another parameter denoted (α) was calculated via the difference between the LUMO energy levels of the studied dyes and the LUMO energy level of PCBM (-3.2 eV) [22]. The value can be obtained by using the following Eq. (4):

4 $$\mathrm{\alpha }=\left|{\mathrm{E}}_{\mathrm{LUMO}}^{\mathrm{acceptor}}\right|- \left|{\mathrm{E}}_{\mathrm{LUMO}}^{\mathrm{donor}}\right|$$

The light-harvesting efficiency (LHE) is obtained using the following equation (LHE = 1-10^f^) [22], where f is the oscillator strength of the dye molecular structure; the calculated LHE is presented in Table 4.

**Table 4:** Calculated electronic absorption parameters for compounds (DTT, BDTTB, BDTTMB and BDTTMP)**.** The CAM-B3LYP functional was applied with a 6-31G +  + (d, p) basis set

Compound	Excited-state	Electronic transition	E_ex_	f	Coefficient
DTT	1	HOMO-1- > LUMO HOMO-2—> LUMO + 2	3.1808 (390 nm)	0.005	0.926 0.035
	2	HOMO-2—> LUMO + 2 HOMO- > LUMO	3.7305 (390 nm)	0.110	0.047 0.928
	5	HOMO-3—> LUMO HOMO-2—> LUMO HOMO—> LUMO + 2	4.3883 (282 nm)	0.1210	0.191 0.541 0.221
BDTTB	1	HOMO-2—> LUMO + 1 HOMO-2—> LUMO + 3	2.976 (416 nm)	0.0063	0.931 0.052
	3	HOMO-2—> LUMO + 1 HOMO-2—> LUMO + 3 HOMO—> LUMO + 1	3.563 (348 nm)	0.0476	0.027 0.040 0.890
	6	HOMO-3—> LUMO + 2 HOMO-3—> LUMO HOMO-1—> LUMO + 2	3.699 (335 nm)	0.0018	0.805 0.020 0.060 0.074
BDTTMB	1	HOMO-3—> LUMO HOMO-2—> LUMO + 1	2.973 (417 nm)	0.0025	0.461 DT0.461
	3	HOMO-3—> LUMO + 3 HOMO-2—> LUMO + 2 HOMO-1—> LUMO + 1 HOMO—> LUMO	3.557 (348 nm)	0.0187	0.020 0.020 0.421 0.467
	5	HOMO-3—> LUMO + 3 HOMO-2—> LUMO + 2 HOMO-1—> LUMO + 1 HOMO—> LUMO	3.638 (341 nm)	0.0014	0.407 0.420 0.028 0.031
BDTTMP	1	HOMO-3—> LUMO + 1 HOMO-2—> HOMO	2.968 (418 nm)	0.001	0.461 0.464
	3	HOMO-1—> LUMO + 1 HOMO—> LUMO	3.475 (357 nm)	0.0024	0.446 0.483
	5	HOMO-3—> LUMO + 2 HOMO-3—> LUMO + 3 HOMO-2—> LUMO + 2 HOMO-2—> 1 LUMO + 3	3.684 (336 nm)	0.0004	0.330 0.125 0.134 0.273

As presented in Table 4, the obtained values of α were in the range of (0.938–1.138 eV). This indicates, that all LUMO levels for all studied compounds are placed higher than the LUMO level of PCBM. So, these triazine derivatives compounds can be utilized as sensitizers of the electron injection process from the excited dye to the conduction band of PCBM. Depending on the values of LHE in Table 4, the best dye that can act as DSSCs is DDT dye compared to the rest compounds under study.

### UV–Vis Properties

**Fig. 5:**
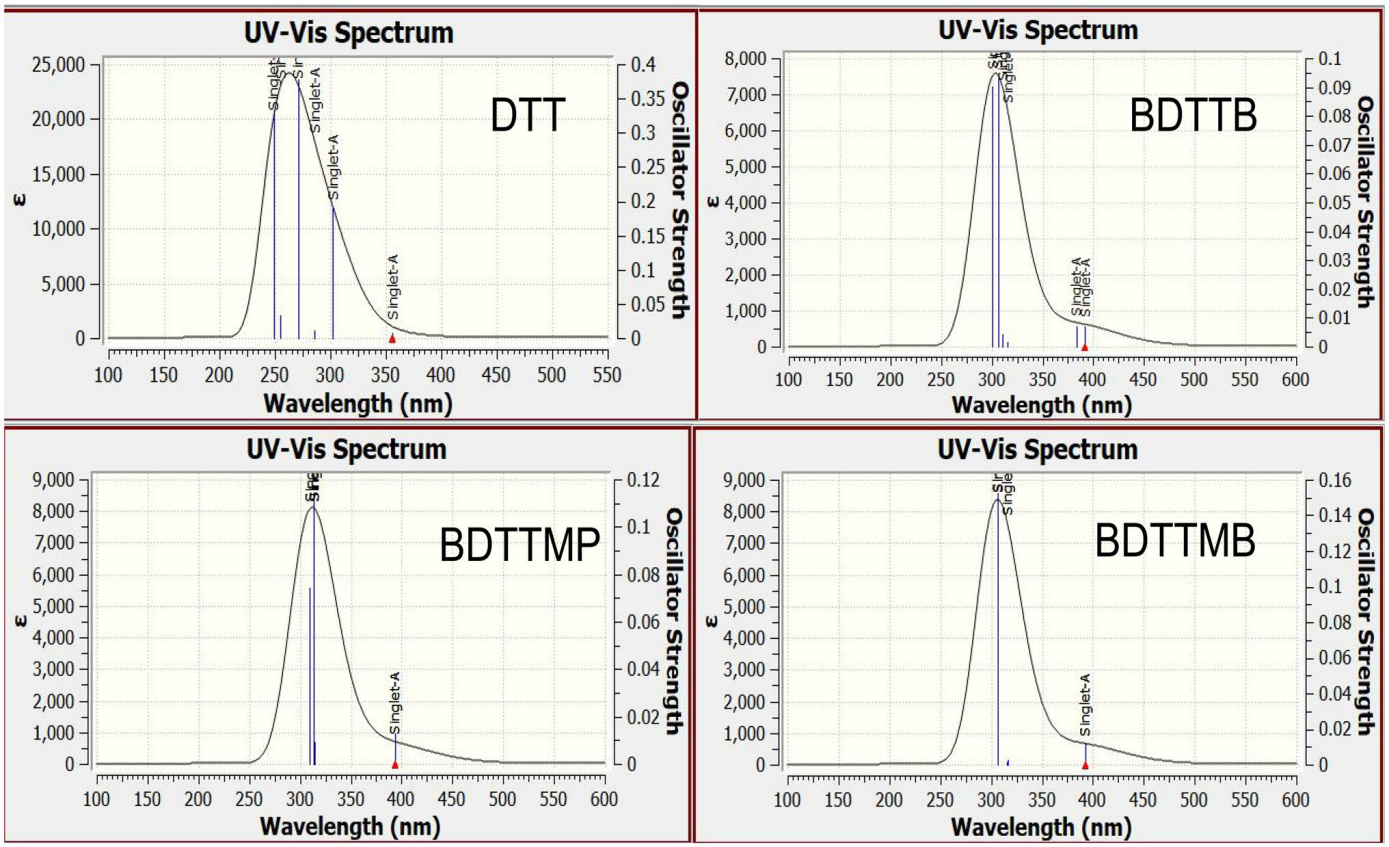
The calculated electronic UV–Vis absorption spectra for all studied molecules (**DTT**, **BDTTB**, **BDTTMB** and **BDTTMP**) in methanol using TD-DFT/ CAM-B3LYP / 6-31G +  + (d, p)

The optical properties and the electronic transition for the all studied dyes (DTT, BDTTB, BDTTMB and BDTTMP) are investigated using TD-DFT/ CAM-B3LYP / 6-31G +  + (d, p) method [23]. The calculated transition energies of studied molecular structures for the absorption wavelength, vertical excitation energy (E_ex_), oscillator strength (f), and the transition character of these dyes are listed in Table 4. The UV–Vis absorption spectra of triazine dye derivatives simulated at the TD-DFT/ CAM-B3LYP / 6-31G +  + (d, p) method are located from 300 to 450 nm as shown in Fig. 5. The experimental electronic absorption spectra of the DDT, BDTTB, BDTTMB, and BDTTMP molecular structures were obtained [17]. The electronic experimental maximum absorption wavelengths were 400, 350, 350, and 350 for DDT, BDTTB, BDTTMB, and BDTTMP molecules respectively. The calculated electronic absorption spectrum of DTT in methanol appears as three transitions at 390, 332, and 382 nm. The first one refers to the experimental peak at 400 nm (0.005) that arises from the transition of HOMO-1—> LUMO. On the other hand, a second band (f = 0.110) arises from a transition of HOMO—> LUMO and the third one (f = 0.1210) due to electronic transition of HOMO-2—> LUMO. Also, the calculated electronic absorption of the other molecular structures (BDTTB, BDTTMB, and BDTTMP) are investigated via using the same level as presented in Fig. 5 and Table 4. The three calculated electronic transitions for BDTTB dye appear at 416 nm, 348 nm and 335 nm in methanol where in agreement with practical results. The first, second and third absorption peak arises from the electronic transition HOMO-2—> LUMO + 1, HOMO—> LUMO + 1 and HOMO-3—> LUMO + 2. The same findings were also found for both BDTTMB and BDTTMP but with red shift for DTT one.

### Natural Bond Orbital (NBO) Analysis

Since resonance is a fundamental phenomenon in the stability of the organic chemical structure, it was essential to study the stability of the studied molecular chemical structures (DDT, BDTTMB, BDTTB, and BDTTMP) using Natural Bond Orbital (NBO) analysis. The hyperconjugation interactions in (DDT, BDTTMB, BDTTB, and BDTTMP) molecular structures are examined via NBO analysis theory utilizing the B3LYP/6-31G(d, p)level of theory [24]. Besides, these results were obtained by utilizing second request annoyance energies (E(2)). 24] The utmost influential second-order perturbation (E(2)) delocalization energies in all studied molecular structures in their gaseous phase are presented in Table 5. These are classified as π- π* and n- π* interactions, with the latter having larger energy magnitudes. The intense interaction between C_2_-N_4_ π-bond to C_1_-N_6_ π-antibonding stabilized DDT molecular structure by 22.84 kcal/mol then from C_3_-N_5_ π-bond to C_4_-N38 π-antibonding via 32.31 kcal/mol. Also, the interaction between C_20_- C_25_ bond and C_23_-C_24_ π-antibonding is stabilized DDT molecule via 21.40 kcal/mol then from C_20_-C_25_ π-bond to C_21_-C_22_ π-antibonding that contributed 19.81 kcal/mol respectively as recorded in Table 5. In addition, the interaction between LP S_7_ to π*_C3—N5_ in DDT that assigned by 21.31 Kal/mol. The delocalization of MO between C_1_-C_6_ π-bond to C_2_-C_3_ and C_4_-C_5_ π-antibonding then from C_4_-C_5_ π-bond to C_1_-C_6_ π-antibonding is stabilized BDTTMB molecular structure via 21.01, 19.70 and 20.54 kcal/mol respectively. Also, the interactions among the following; (π_C15—N21_ and π*_C17—N23_, π_C17—N23_ and π*_C16—N22_, π_C16—N22_ and π*_C15—N21_, π_C19—N24_ and π*_C18—N25_, π_C28—C30_ and π*_C29—C31_ and LP S_14_ and π*_C15—N21_) are stabilized BDTTB by 21.03, 19.46, 30.86, 31.96, 19.60 and 27.17 kcal/mol respectively. Finally, the stability of BDTTMP molecular structure due to the interaction between π-bond and π-antibonding; the values of interactions are listed in Table 5.

**Table 5:** Selection of most influential order perturbation estimation of the hyper conjugative energies (Kcal/mol) of DDT, BDTTMB, BDTTB, and BDTTMP triazine derivatives compounds utilizing the DFT/B3LYP/6-31G (d, p) method

DDT		BDTTMB		BDTTB		BDTTMP	
Interaction	Energy (Kcal/mol)	Interaction	Energy (Kcal/mol)	Interaction	Energy (Kcal/mol)	Interaction	Energy (Kcal/mol)
π_C2–N4_ to π*_C1—N6_	14.02	π_C1—C6_ to π*_C2—C3_	21.01	π_C15—N21_ to π*_C17—N23_	21.03	π_C1—C2_ to π*_C3—C4_	22.23
π_C2–N4_ to π*_C3—N5_	32.31	π_C1—C6_ to π*_C4—C5_	19.70	π_C16—N22_ to π*_C15—N21_	30.86	π_C1—C2_to π*_C5—N6_	17.16
π_C3—N5_ to π*_C1—N6_	21.32	π_C4—C5_ to π*_C1—C6_	20.54	π_C17—N23_ to π*_C16—N22_	19.46	π_C3—C4_ to π*_C5—N6_	28.43
π_C3—N5_ to π*_C2—N4_	11.65	π_C19—N26_ to π*_C21—N25_	20.44	π_C19—N24_ to π*_C18—N25_	31.96	π_C18—N24_ to π*_C20—N25_	22.54
π_C20—C25_ to π*_C21—C22_	19.81	π_C20—N27_ to π*_C19—N26_	31.41	π_C20—N26_ to π*_C19—N24_	21.10	π_C19—N26_ to π*_C18—N24_	32.16
π_C20—C25_ to π*_C23—C24_	21.40	π_C21—N25_ to π*_C20—N27_	21.28	π_C28—C30_ to π*_C29—C31_	19.60	π_C20—N25_ to π*_C19—N26_	21.12
LPS_7_ to π*_C3—N5_	21.32	LPS_18_ to π*_C22—N28_	24.34	LPS_14_ to π*_C15—N21_	27.17	LPS_17_ to π*_C21—N27_	25.11

### Molecular Electrostatic Potentials Map (MEPM)

**Fig. 6:**
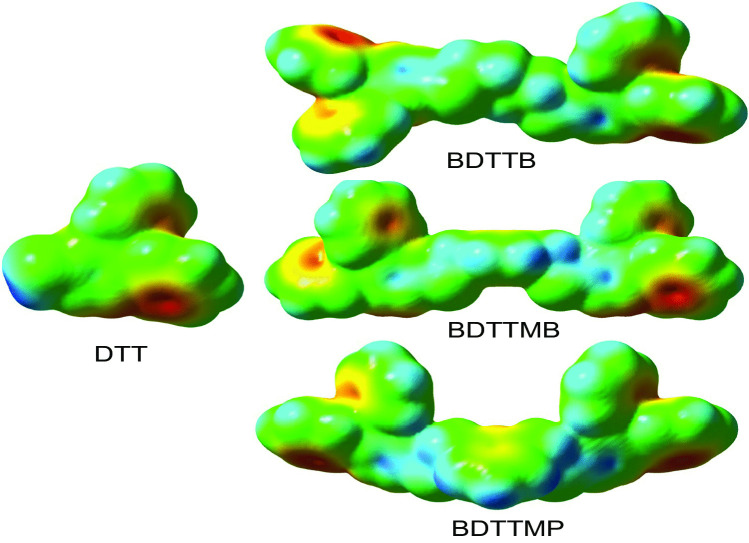
Electrostatically mapped surfaces of **DTT**, **BDTTB**, **BDTTMB**, and **BDTTMP** compounds

A molecular electrostatic potential map (MEPM) investigates reveals the electrophilic versus nucleophilic sites of all molecular structures under study, giving for the prediction of reaction areas [25]. The MEPMs of the studied DTT and its derivatives were calculated employing B3LYP/6-31G (d,p) in Fig. 6. The negatively, positively and neutral zones of the MEPM interfaces are represented by the colors red, blue, and green, respectively (Fig. 6). The negative site (red color) is inside terminal benzene rings, suggesting that such rings are electrophilic attacks. The positive area (blue color) encompassed the carbon and hydrogen atoms, suggesting nucleophilic attacks in these sites. The neutral areas ( green color) of the molecular structure are those that are devoid of any charge distribution. The blue area around the H-atoms of the sulfur atom was associated with electron deficiency (i.e., nucleophilic reactivity), rendering it a favorable place for intermolecular H-bonding (nucleophile attack). The electron density (red area) appeared centered within the circle, according to the ESP map. The nucleophile in the phenyl ring has had the highest negative possibility, making it an ideal subject for an electrophilic engagement [26].

### Evaluating DTT Dye for DSSC Device

**Fig. 7:**
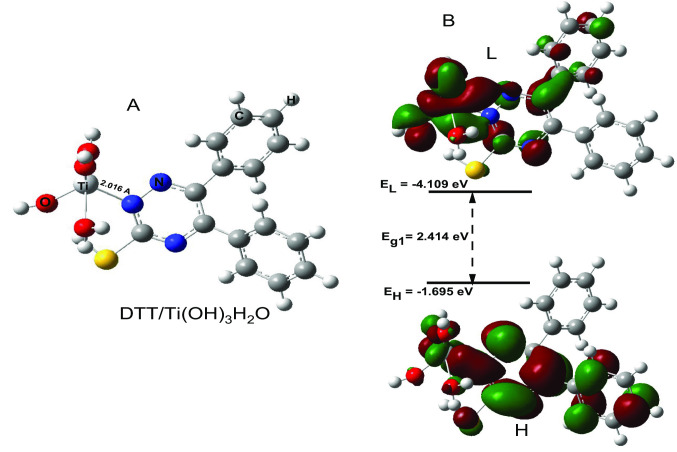
DTT/Ti(OH)_3_H_2_O optimized molecular structure (**A**). H and L Molecular orbitals as well as the energy gap between H and L (E_g1_) (**B**). DFT/B3LYP/LANL2DZ method was used to obtain DTT/Ti(OH)_3_H_2_O, H, and L MOs optimized molecular structure

we continue our study of a prototypical dye molecule for DSSC devices from Fig. 7. In an actual device, the DTT end of the dye molecule is bound to a titanium oxide nanostructure. The original researchers [27] validated that the metal surface, in this case, can be adequately represented as a Ti(OH)_3_H_2_O moiety: the titanium atom is an octahedral complex with two positions taken up by the oxygen atoms of the acrylic acid anchor [27], three hydroxyl groups and one water molecule. Because of the high stability of the DTT compound compared to the remaining compounds under study, the DTT compound was chosen as a representative to study the DTT complexes with the Ti(OH)_3_H_2_O. The DTT/ Ti(OH)_3_H_2_O optimized structure, HOMO and LUMO MOs, and energy gap (E_g1_) between HOMO and LUMO were obtained using DFT/B3LYP/LANL2DZ method and the calculated results are shown in Fig. 7(A and B). The nitrogen atom of the pyrazine ring that is present in DTT dye is linked to the titanium atom of Ti(OH)_3_H_2_O through a coordinated bond as shown in Fig. 7. The N → Ti coordination bond length is 2.016 A (see Fig. 7A), this is confirming the stability of the DTT/Ti(OH)_3_H_2_O complex. The fact that it is a charge-transfer state is again evident. Electrons are moving from the DTT benzene end to the other benzene end of the DTT molecule as shown in Fig. 7B. Interestingly. The titanium complex is also involved in HOMO and LUMO, hence it is becoming part of the donor and acceptor. The 3d orbital from the titanium atom is visible in the LUMO and HOMO, indicating that electrons in the Ti complex interfere with the electronic transition. They receive and donate electrons.

## Conclusion

In this paper, the optimized molecular structure, and electronic and optical properties of four triazine derivative dyes DTT, BDTTB, BDTTMB, and BDTTMP were investigated via utilizing DFT/TD-DFT. According to the ground state geometry, we note that all stable conformations are not planar. The HOMO/LUMO energy gaps of DTT, BDTTB, BDTTMB and BDTTMP were calculated at DFT/B3LYP/6-31G (d, p) level are 4.168, 3.528, 3.965 and 3.921 eV respectively. So, the energy gaps differ slightly and decrease in the following order: BDTTB < BDTTMP < BDTTMB < DTT. Consequently, the calculated values of Voc/ TiO2 of our dyes are sufficient for possible efficient electron injection from the donor to the acceptor.

## Data Availability

All data generated or analyzed during this study are included in this published article.
